# A novel circular RNA circ-LRIG3 facilitates the malignant progression of hepatocellular carcinoma by modulating the EZH2/STAT3 signaling

**DOI:** 10.1186/s13046-020-01779-5

**Published:** 2020-11-23

**Authors:** Suofeng Sun, Jing Gao, Shen Zhou, Yuan Li, Yu Wang, Li Jin, Jian Li, Bowei Liu, Bingyong Zhang, Shuangyin Han, Hui Ding, Xiuling Li

**Affiliations:** 1 Department of GastroenterologyHenan Provincial People’s Hospital, People’s Hospital of Zhengzhou University, School of Clinical Medicine, Henan University, Zhengzhou, 450003 Henan China; 2 Department of Microbiome Laboratory, Henan Provincial People’s Hospital, People’s Hospital of Zhengzhou University, Zhengzhou University, Zhengzhou, China; 3 Department of Traditional Chinese Medicine, The Third Affiliated Hospital Affiliated of Henan University of Traditional Chinese Medicine, Zhengzhou, 450003 Henan China; 4 Xinxiang Medical University, Xinxiang, 453000 Henan China; 5 Xinxiang aviation industry (Group) Co., Ltd. Hospital, Xinxiang, 453000 Henan China

**Keywords:** Circular RNA, Hepatocellular carcinoma, Biomarker, STAT3 signaling, Molecular interaction

## Abstract

**Background:**

Circular RNA (circRNA) is emerging as an important player in human diseases, especially cancer. In our previous study, we identified a series of deregulated circRNAs in hepatocellular carcinoma (HCC) by performing circRNA microarray expression profile. Here, we aimed to explore the role of circ-LRIG3 (hsa_circ_0027345) in HCC.

**Methods:**

qRT-PCR and western blot were used to asses gene and protein expression, respectively. CCK-8, EdU and Transwell assays were used to detect cell proliferation, migration and invasion. GSEA software was applied to analyze the pathway related to circ-LRIG3. Co-IP, RIP and ChIP assays were used to identify the positive feedback axis of circ-LRIG3/EZH2/STAT3. Animal study was carried to test the role of circ-LRIG3 in vivo.

**Results:**

Circ-LRIG3 was notably upregulated in HCC and promoted HCC cell proliferation, migration, invasion and reduced apoptosis. Circ-LRIG3 formed a ternary complex with EZH2 and STAT3, facilitating EZH2-induced STAT3 methylation and subsequent phosphorylation, resulting in the activation of STAT3 signaling. In turn, activated STAT3 could directly bind to circ-LRIG3 promoter to increase circ-LRIG3 transcription activity, thus forming a positive feedback loop. The animal models showed that exogenous expression of circ-LRIG3 enhanced tumorigenicity and metastasis in vivo, whereas these effects were blocked after treatment with C188–9, a specific STAT3 small-molecule inhibitor. Clinically, high circ-LRIG3 was closely linked with aggressive clinicopathological features and was identified as an independent risk prognostic factor of overall survival. Importantly, plasma circ-LRIG3 was found to be a highly sensitive and specific non-invasive diagnostic indicator for HCC.

**Conclusions:**

Our study reveals the carcinogenic role of circ-LRIG3 in HCC, which may provide a new therapeutic target for HCC patients.

**Supplementary Information:**

The online version contains supplementary material available at 10.1186/s13046-020-01779-5.

## Background

Hepatocellular carcinoma (HCC) is the main histological subtype of primary liver cancer with rapidly increasing morbidity and mortality, especially in China [1]. Although many patients are effectively diagnosed and treated in the early stage of the disease, the recurrence rate is still very high [2, 3]. And the prognosis of HCC is not optimistic, especially for patients with advanced clinical stage, when tumor spreads to the surrounding lymph nodes, the 5-year survival rate of patients is only 11%; when cancer spreads to some important organs, the situation is worse, with a 5-year survival rate of only 3% [4]. Therefore, dissecting mechanisms underlying HCC will yield new directions in clinical diagnosis and treatment for the benefit of patients.

Circular RNA (circRNA) is a new kind of special non coding RNA (ncRNA) molecule, which is the latest research hotspot in RNA field. Different from the traditional linear RNA, circRNA has a closed loop structure, which is not affected by RNA exonuclease, and is more stable and not easy to degrade [5]. The classical RNA sequencing method can only separate RNA with characteristic “poly-A tail”, thus circRNA is discarded in the classical separation process due to the link between the head and tail, this is the reason why circRNA has long been ignored by scientists. With the advent of high-throughput sequencing, numerous circRNAs has been found and they are produced from special selective back splicing of pre-mRNA and are abundant in the eukaryotic cells [6–8]. CircRNA is highly conservative and expressed in a tissue and developmental-stage specific pattern [9].

The mechanisms by which circRNA works in cell fate are only beginning to be elucidated, and most researchers have focused on circRNA as a sponge of miRNA molecules [10]. In this functional model, circRNA effectively sponges miRNAs to elevate their targets, indirectly regulating gene expression [11]. The most known sample is CDR1as, which harbors more than 70 conserved miR-7 binding sites [12, 13]. To date, only a small portion of researchers have focused on the direct interactions between circRNA and proteins [14]. For instance, circ-AMOTL1 was reported to be physically interacted with c-Myc, facilitating c-Myc nuclear translocation, resulting in upregulating a cohort of c-Myc targets [15]. Circ-CUX1 could directly bind EWSR1 and enhance its interaction with MAZ, leading to MAZ transactivation and neuroblastoma progression [16]. And our research team found that circ-ADD3 increased the interaction between CDK1 and EZH2, resulting in EZH2 phosphorylation and ubiquitination and degradation, inhibiting HCC metastasis [17]. These studies suggest that the circRNA-protein interaction model plays a fundamental role in cancer cell biology.

In our previous circRNA microarray study, we found a series of aberrantly expressed circRNAs in HCC [17], including circ-LRIG3 (hsa_circ_0027345, chr12: 59277301–59,308,117), a circRNA derived from the back-splicing of LRIG3 exon 2 ~ 11. Here, we aimed to delineate the biological function, clinical significance and potential mechanism of action of circ-LRIG3 in HCC.

## Materials and methods

### Chemicals and antibodies

RNase R was purchased from Epicentre (Farmingdale, NY, USA), actinomycin D purchased from RayStarBio (HangZhou, Zhejiang, China) was used to assess the half-life. The specific STAT3 small-molecule inhibitor C188–9 was obtained from StemMed, Ltd. (Monmouth, NJ, USA). And the STAT3 activator colivelin was purchased from Santa Cruz Biotechnology (sc-361,153). The primary antibodies including anti-p-STAT3 (#9145), anti-STAT3 (#9139), anti-EZH2 (#5246), anti-GAPDH (#2118) and anti-Ki-67 (#9449) were all purchased from Cell Signaling Technology (Danvers, MA, USA).

### HCC tissues, plasma and cell lines

A total of 130 paired surgically excised HCC and adjacent normal tissues from Henan Provincial People’s Hospital (Zhengzhou, China) were immediately collected into liquid nitrogen to preserve the integrity of the RNA. In addition, plasma samples from 36 HCC patients and 36 healthy controls were also collected to test the non-invasive diagnostic value of circ-LRIG3. None of the patients received preoperative radiotherapy or chemotherapy, and they all provided written informed consent. This study was conducted with the approval of the ethics committee of Henan Provincial People’s Hospital. Six HCC cell lines including SNU-423, HepG2, Hep3B, Huh7, SMMC-7721 and MHCC-97 L and one normal LO2 hepatocytes were all purchased from ATCC and cultured in DMEM medium containing penicillin/streptomycin and 10% fetal bovine serum.

### RNA isolation and qRT-PCR analysis

Total RNA was classically extracted by Trizol reagent (Invitrogen, CA, USA) and dissolved in DEPC water. Then, RNA was reverse transcripted into cDNA using All-in-One™ First-Strand cDNA Synthesis Kit (GeneCopoeia, MD, USA), followed by quantification using BlazeTaq™ SYBR Green qPCR Mix 2.0 (GeneCopoeia). The primer sequences are shown in Table S3. GAPDH was used as the internal reference. The relative expression was calculated using the comparative Ct (2^-ΔΔCt^) method. For determining the junction sequence of circ-LRIG3, the PCR product was purified and subjected to Sanger sequencing.

### Fluorescence in situ hybridization (FISH)

The FISH assay was performed by using RNA FISH Kit (GenePharma, Shanghai, China) according to manufacturer’s protocol with FAM-labeled circ-LRIG3 probe against the junction site of circ-LRIG3 (GenePharma). The nucleus was stained with DAPI. The fluorescence signal was observed, photographed and merged using confocal laser microscope system.

### Generation of circ-LRIG3-overexpressing and -silenced HCC cell lines

To overexpress circ-LRIG3, pLC5-ciR-GFP (#GS0108) vector containing one paired reverse complementary sequence (RCS) provided by Geneseed (Guangzhou, China) was used, in which the full-length of circ-LRIG3 (chr12: 59277301–59,308,117) was inserted into pLC5-ciR vector between EcoRI and BamHI restriction sites, followed by packaging into the lentivirus and infection into HepG2 cells. The stable circ-LRIG3-overexpressing cells were selected by 0.8 μg/mL puromycin. To knock down circ-LRIG3, two antisense oligonucleotide (ASO) against circ-LRIG3 junction site were designed and synthesized by RiboBio (Guangzhou, China). Then, the ASO was transfected into SMMC-7721 cells using riboFECT™ CP transfection solution (RiboBio) based on manufacturer’s protocol.

### Cell proliferation and apoptosis assays

The CCK-8 assay was used to assess cell viability. In brief, 1500 HCC cells were seeded into the 96-well plate. After 24, 48 and 72 h, the medium was added with 10 μL CCK-8 solution (Dojindo, Kumamoto, Japan) for 2.5 h, followed by detection of the absorption value using a microplate spectrophotometer. Besides, the DNA synthesis rate was assessed by using EdU Staining Proliferation Kit provided by RiboBio based on manufacturer’s protocol. The positive cells were observed and photographed using confocal laser microscope system. For detecting cell apoptosis, the Annexin V-PE/7-ADD Apoptosis Detection Kit (BD Biosciences, CA, USA) was used, in which HCC cells were incubated with 5 μL Annexin V-PE and 5 μL 7-ADD in 500 μL binding buffer, followed by analyzing apoptotic cells using a flow cytometer (BD Biosciences).

### In vitro invasion and migration assays

The upper chamber of the trans-well insert with pore size of was coated with (invasion) or without (migration) Matrigel (Corning) and was placed in a 37 °C incubator for 2.5 h. Then, HCC cells were added into the upper chamber, and the lower chamber was added with 600 μL DMEM complete medium. 20 h later, the trans-well was fixed with formaldehyde for 5 min, permeabilized by methanol for 5 min, and was then stained by 0.5% crystal violet for 10 min. The invaded/migratory cells were counted under a light microscope at least 5 random fields.

### mRNA sequencing

SMMC-7721 cells were transfected with control and two ASOs (ASO-circ-LRIG3#1 and #2) for 48 h, then cells were washed and total RNA was extracted by TRIzol and purified by DNase I. After that, the oligo-dT magnetic beads were used to separate mRNA from total RNA for construction of cDNA libraries (GeneSky, Shanghai, China). Lastly, the differentially expressed genes (fold change ≥2 and *P* < 0.05) were determined and subjected to Gene Set Enrichment Analysis (GSEA). The RNA sequencing data was deposited in the Gene Expression Omnibus database under accession code GSE154021.

### Western blot, co-IP and immunohistochemistry (IHC)

For Western blot, whole cell extract was prepared by lysing cells in NP-40 lysis buffer. Then, proteins were resolved by 10% SDS-PAGE gel and transferred onto PVDF membrane. After incubating with corresponding primary and secondary antibodies, the membrane was strictly washed using TBST, and the chemiluminescent signals were developed using Clarity™ Western ECL Substrate (Bio-Rad, CA, USA). The Co-IP assay was carried out as previously described [17]. The IHC assay was performed by using Histostain-Plus IHC Kit (NeoBioscience, Guangzhou, China) as per Manufacturer’s instructions.

### RNA pull-down and RIP assays

For the RNA pull-down assay, the biotinylated probe against circ-LRIG3 junction site was designed and synthesized by RiboBio. Then, the indicated HCC cell lysates were prepared using the NP-40 lysis buffer and incubated with above probe at 26 °C for 2 h with agitation. 50 μL streptavidin magnetic beads (Invitrogen) were pre-washed with 20 mM Tris (pH = 7.5) twice and added into above complex, incubating at 26 °C for 30 min with agitation. After strict washing, the proteins enriched by circ-LRIG3 were eluted for Western blot analysis. The RIP assay was performed using Magna^RIP^ RNA-Binding Protein Immunoprecipitation Kit (Millipore, MA, USA) based on the manufacturer’s instructions with additional UV crosslink step as previously described [17].

### Chromatin immunoprecipitation (ChIP)

HCC cells were treated with formaldehyde for 10 min, followed by addition with glycine for 5 min. Then, cells were harvested and digested with a micrococcal nuclease for 20 min at 37 °C, making DNA break into 200 ~ 1000 bp fragments. The indicated antibodies were incubated with above cell lysates at 4 °C overnight with agitation. The second day, the protein A agarose (Santa Cruz Biotechnology) was prepared and added into above antibody-target protein-DNA complex, and incubated for 1 h at 26 °C with agitation. Lastly, the enriched DNA fragments were purified and collected for PCR analysis.

### Luciferase reporter assay

The Cignal STAT3 Reporter (luc) Kit (SA Biosciences, CA, USA) was used to assess STAT3 transcriptional activity according to manufacturer’s instructions. For determining the effect of STAT3 on transcriptional activity of circ-LRIG3 promoter, the full-length of circ-LRIG3 promoter with wild-type or mutant STAT3 binding site was inserted into pGL3-basic vector (Promega, WI, USA), followed by transfection into HepG2 and SMMC-7721 cells treated with colivelin or c188–9. The Dual-Luciferase Reporter Assay System (Promega) was applied to detect luciferase luminescence.

### Animal experiments

The stable circ-LRIG3-overexpresing HepG2 cells were subcutaneously or tail vein injected into age-matched male BALB/c mice, followed by growth for 5 weeks under specific-pathogen-free conditions. During this period, the mice were intraperitoneally injected with C188–9 (50 mg/kg) daily for 3 weeks. At the end of observation, the mice were killed by cervical dislocation, and the subcutaneous tumors were weighed and subjected to hematoxylin&eosin (H&E), IHC and TUNEL (Beyotime, Beijing, China) staining. For mice injected with cells via the tail vein, the lung tissues were collected for counting surface metastases under a dissecting microscope, followed by H&E staining. All experimental procedures in our study were approved by the Institutional Animal Care and Use Committee of Henan Provincial People’s Hospital.

### Statistics

Comparisons between two groups were analyzed by Student’s t test or Chi-square test as appropriate. ROC and Kaplan Meier-plotter were used to determine the diagnostic and prognostic values of circ-LRIG3, respectively. The link between circ-LRIG3 and p-STAT3 expression in HCC tissues was analyzed by Pearson Correlation Coefficient. All *P*-values were two-sided unless otherwise specified. *P* < 0.05 is indicative of statistical significance.

## Results

### Circ-LRIG3 is highly expressed in HCC and preferentially located in the nucleus

**Fig. 1:**
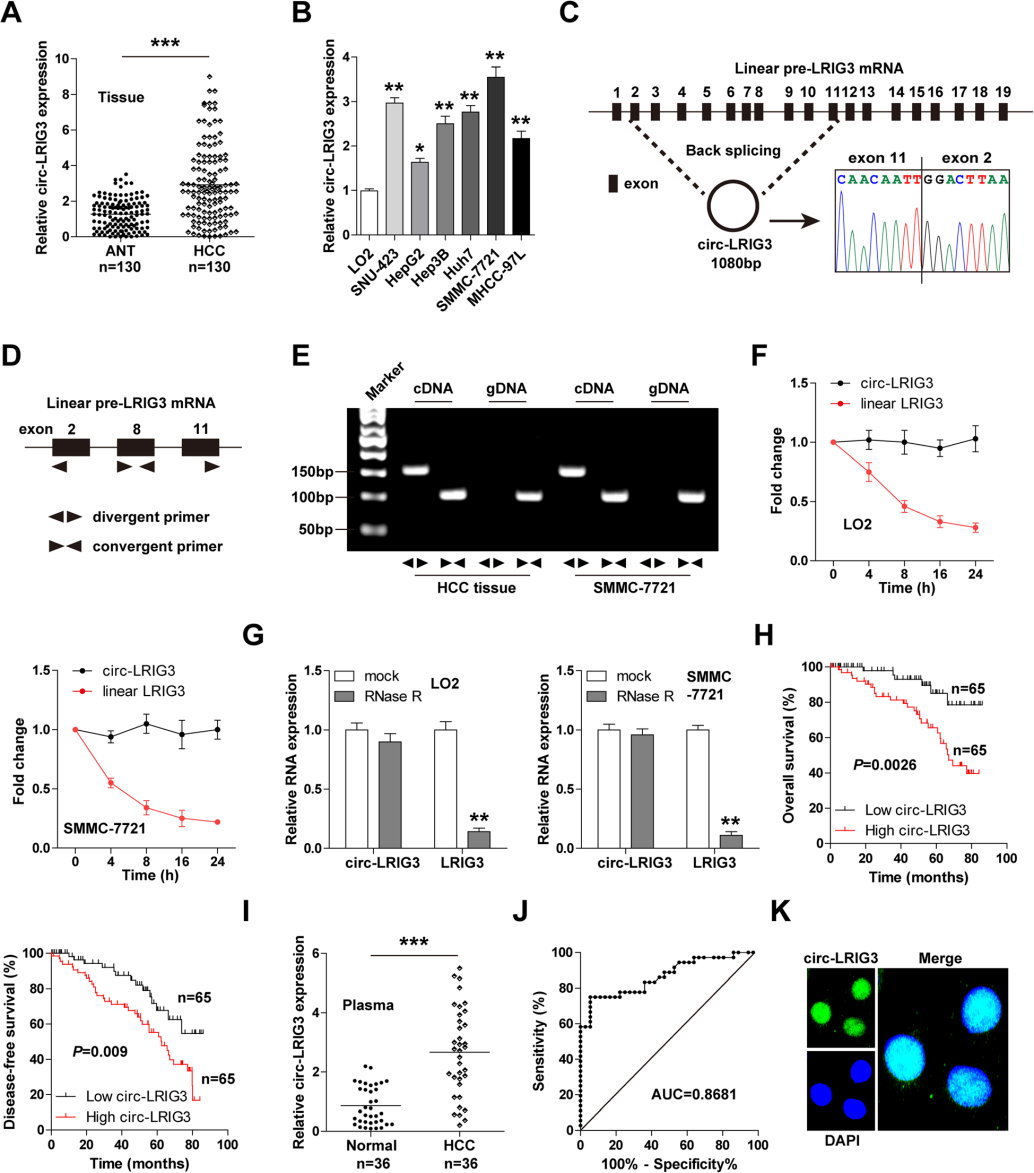
Circ-LRIG3 is upregulated in HCC tissues, cells and plasma. **a**, **b** qRT-PCR analysis of circ-LRIG3 expression in adjacent normal tissues (ANT), HCC tissues and cell lines. **c** The diagrammatic sketch showing the source of circ-LRIG3, and this was confirmed by Sanger sequencing. **d** The schematic diagram of design of divergent and convergent primers. **e** PCR analysis using designed divergent and convergent primers in cDNA and gDNA from HCC tissue and SMMC-7721 cells. **f**, **g** qRT-PCR analysis of circ-LRIG3 and linear LRIG3 expression in LO2 and SMMC-7721 cells after treatment with actinomycin D or RNase R. (H) The overall and disease-free survival curves of HCC patients with low and high circ-LRIG3 expression. **i** qRT-PCR analysis of circ-LRIG3 in plasma from HCC and healthy controls. **j** ROC determining the non-invasive diagnostic value of circ-LRIG3 in HCC based on plasma circ-LRIG3 expression. (K) FISH detecting the subcellular localization of circ-LRIG3 in HCC cells. *P < 0.05, **P < 0.01,***P < 0.001

We collected 130 pairs of HCC and paracancerous normal tissues to conduct qRT-PCR. The results showed that circ-LRIG3 was significantly upregulated in HCC (Fig. 1a). Similarly, increased expression of circ-LRIG3 was observed in HCC cells in comparison to human normal LO2 hepatocytes (Fig. 1b). Sanger sequencing verified that circ-LRIG3 is produced by the back splicing of pre-LRIG3 mRNA exon 2 ~ 11, and its mature full-length is 1080 bp (Fig. 1c). Then, we designed divergent and convergent primers to amplify circ-LRIG3 and linear LRIG3, respectively (Fig. 1d). As shown in Fig. 1e, circ-LRIG3 was only amplified in cDNA, but not in gDNA. Further, we treated LO2 and SMMC-7721 cells with 5 μg/ml actinomycin D, and found that circ-LRIG3 had a half-life of more than 24 h (Fig. 1f). Besides, circ-LRIG3, not linear LRIG3, was highly resistance to RNase R (Fig. 1g). These suggest that circ-LRIG3 is a bona fide circRNA. Clinically, HCC patients with high circ-LRIG3 expression had larger tumor size, more vascular invasion, higher edmondson’s grade and later TNM stage than patients with low circ-LRIG3 expression (Table S1). Moreover, high circLRIG3 was positively correlated with short overall and disease-free survival time (Fig. 1h), and it was identified as an independent risk prognostic factor (Table S2). Of note, the qRT-PCR results showed that circLRIG3 was also increased in HCC plasma (Fig. 1i), and the area under the ROC curve (AUC) was 0.8681 (95%CI: 0.7843 ~ 0.9519) (Fig. 1j), implying that circLRIG3 is an excellent indicator for HCC diagnosis. The FISH assay showed that circLRIG3 was mainly localized in the nucleus (Fig. 1k).

### Circ-LRIG3 promotes HCC cell malignant phenotype

**Fig. 2:**
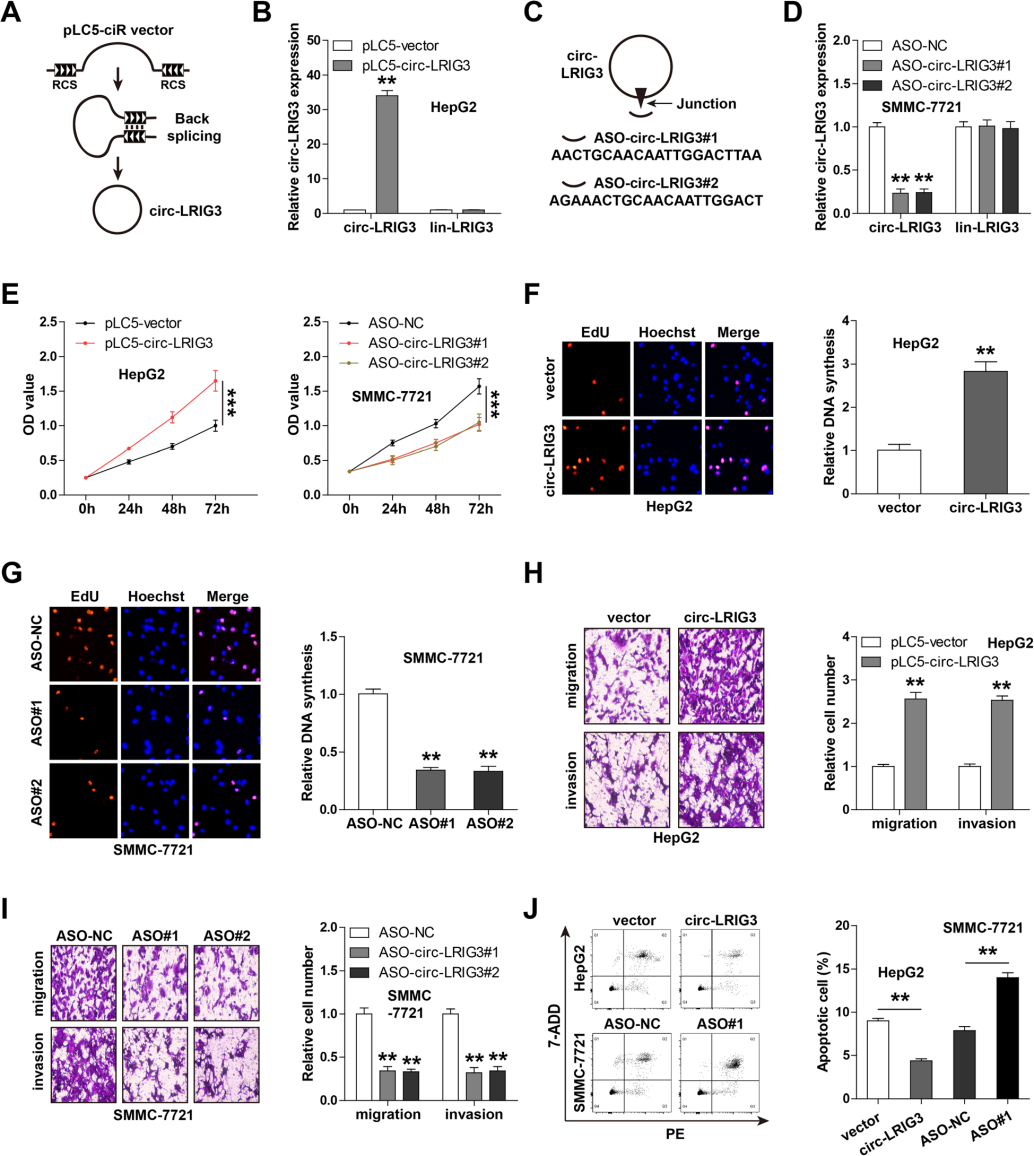
Circ-LRIG3 enhances HCC aggressive phenotype. **a**, **b** The diagrammatic sketch showing the circ-LRIG3-overexpressing pLC5-ciR vector, the overexpression efficiency was verified by qRT-PCR. **c**, **d** The designed ASO against circ-LRIG3 junction site inhibiting circ-LRIG3 expression, the knockdown efficiency was verified by qRT-PCR. **e**-**i** CCK-8, EdU, Transwell migration and invasion assays in circ-LRIG3-overexpressing HepG2 and circ-LRIG3-silenced SMMC-7721 cells. **j** Circ-LRIG3-overexpressing HepG2 and circ-LRIG3-silenced SMMC-7721 cells were stained with PE and 7-ADD dyes, followed by flow cytometry analysis. **P < 0.01,***P < 0.001

To investigate the biological function of circ-LRIG3 in HCC cells, we stably overexpressed circ-LRIG3 in HepG2 cells relatively lowly expressing circ-LRIG3 by using pLC5-ciR lentiviral vector (Fig. 2a, b). And we knocked down circ-LRIG3 in SMMC-7721 cells relatively highly expressing circ-LRIG3 by using antisense oligonucleotide (ASO) targeting the junction site of circ-LRIG3 (Fig. 2c, d). Neither pLC5-circ-LRIG3 nor ASO-LRIG3 affected linear LRIG3 expression (Fig. 2b, d). The CCK-8 results showed that overexpression of circ-LRIG3 increased HepG2 cell viability, while knockdown of circ-LRIG3 decreased SMMC-7721 cell viability (Fig. 2e). Likewise, more DNA synthesis was observed in circ-LRIG3-overexpressing HepG2 cells (Fig. 2f), while less DNA synthesis was observed circ-LRIG3-silenced SMMC-7721 cells (Fig. 2g) as compared with their respective control cells. The migration and invasion of cells were enhanced after ectopic expression of circ-LRIG3 (Fig. 2h), and circ-LRIG3 depletion resulted in an opposite effect (Fig. 2i). In addition, the flow cytometry results showed that circ-LRIG3 overexpression reduced the number of apoptotic cells, whereas circ-LRIG3 depletion increased the number of apoptotic cells (Fig. 2j). More importantly, we found that overexpression of circ-LRIG3 in normal LO2 cells could also increase cell viability and invasion (Fig. S1). These above functional data indicate that circ-LRIG3 is a carcinogenic circRNA in HCC.

### Circ-LRIG3 activates STAT3 signaling pathway in HCC

**Fig. 3:**
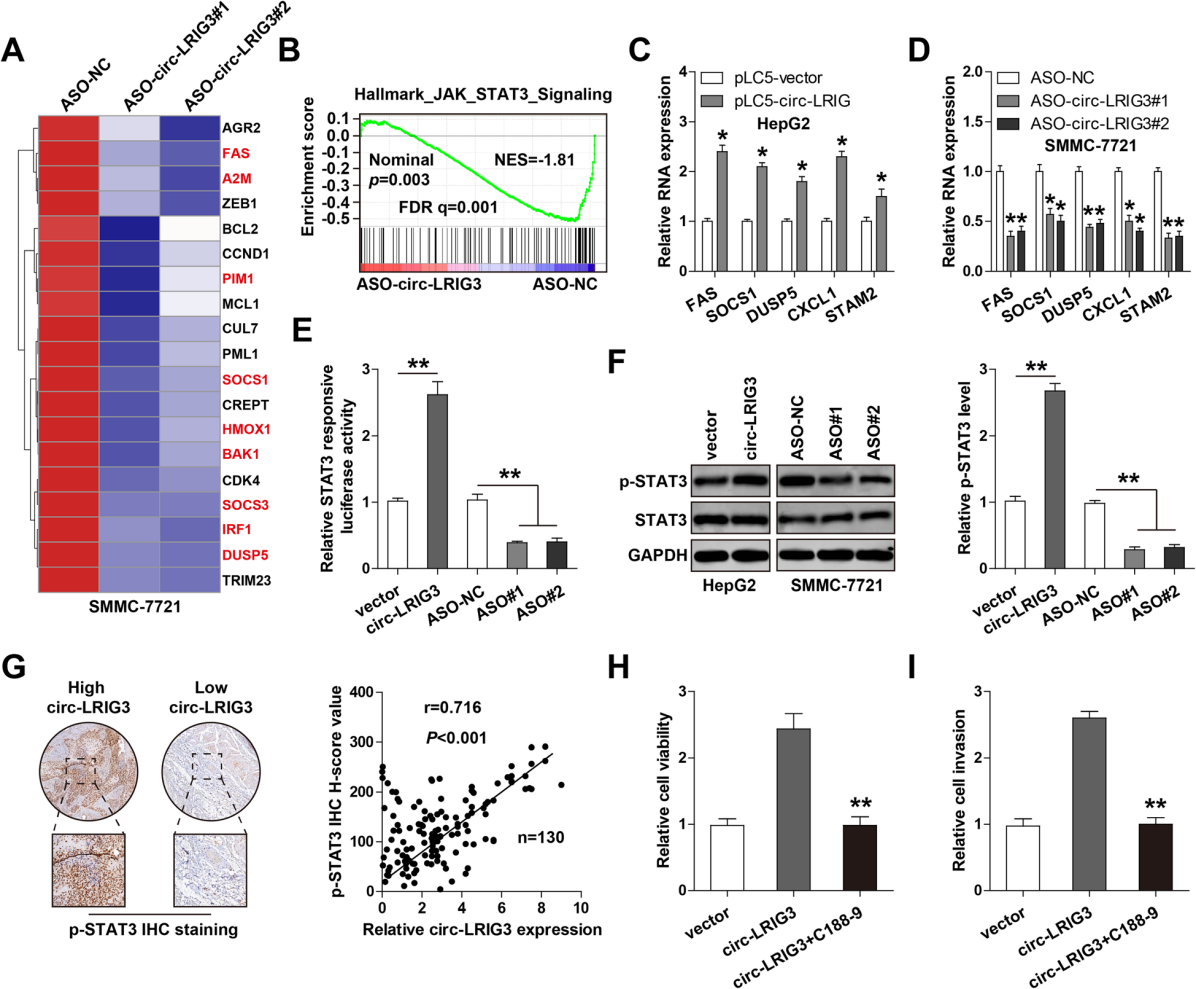
Circ-LRIG3 activates STAT3 signaling in HCC cells. **a**, **b** mRNA sequencing in circ-LRIG3-silenced SMMC-7721 cells, followed by GSEA analysis the enrichment of STAT3 pathway. **c**, **d** qRT-PCR analysis of the indicated gene expression in circ-LRIG3-overexpressing HepG2 and circ-LRIG3-silenced SMMC-7721 cells. **e** Luciferase reporter assay detecting the effect of circ-LRIG3 on the activity of STAT3 pathway. **f** Western blot analysis of p-STAT3 and STAT3 protein levels in circ-LRIG3-overexpressing HepG2 and circ-LRIG3-silenced SMMC-7721 cells, GAPDH was used as a loading control. **g** The correlation between circ-LRIG3 and p-STAT3 in 130 HCC tissues. **h** CCK-8 and Transwell assays detecting the cell viability and invasion in circ-LRIG3-overexpressing HepG2 cells treated with C188–9. *P < 0.05, **P < 0.01

To determine how circ-LRIG3 promotes HCC progression, we performed RNA sequencing in control and circ-LRIG3-silenced SMMC-7721 cells, and found that circ-LRIG3 significantly affected the downstream genes of STAT3 signaling (Fig. 3a). The gene-set enrichment analysis (GSEA) results confirmed the the close connection between circ-LRIG3 and STAT3 signaling (Fig. 3b). Then, we performed qRT-PCR assay to verify RNA sequencing results, as shown in Fig. 3c, the levels of FAS, SOCS1, DUSP5, CXCL1 and STAM2 were notably increased in circ-LRIG3-overexpressing HepG2 cells in comparison to control cells, and depletion of circ-LRIG3 in SMMC-7721 cells led to an opposite trend (Fig. 3d). Further, overexpression of circ-LRIG3 markedly increased, while knockdown of circ-LRIG3 reduced STAT3 transcriptional activity (Fig. 3e). Consistently, the phosphorylation level of STAT3 (p-STAT3) significantly increased after circ-LRIG3 overexpression, and decreased after circ-LRIG3 silencing, but the total STAT3 level remained unchanged (Fig. 3f). Besides, we performed p-STAT3 immunohistochemistry (IHC) staining in HCC tissues, and found that its level was strongly positively associated with circ-LRIG3 level (r = 0.716) (Fig. 3g). Functionally, when HepG2 cells were treated with C188–9, a specific STAT3 small-molecule inhibitor, the enhanced cell viability and invasion caused by circ-LRIG3 overexpression were effectively abolished (Fig. 3h, i). These findings demonstrate that circ-LRIG3 functions via activating STAT3 signaling in HCC.

### Circ-LRIG3 enhances EZH2-mediated STAT3 activation in HCC

**Fig. 4:**
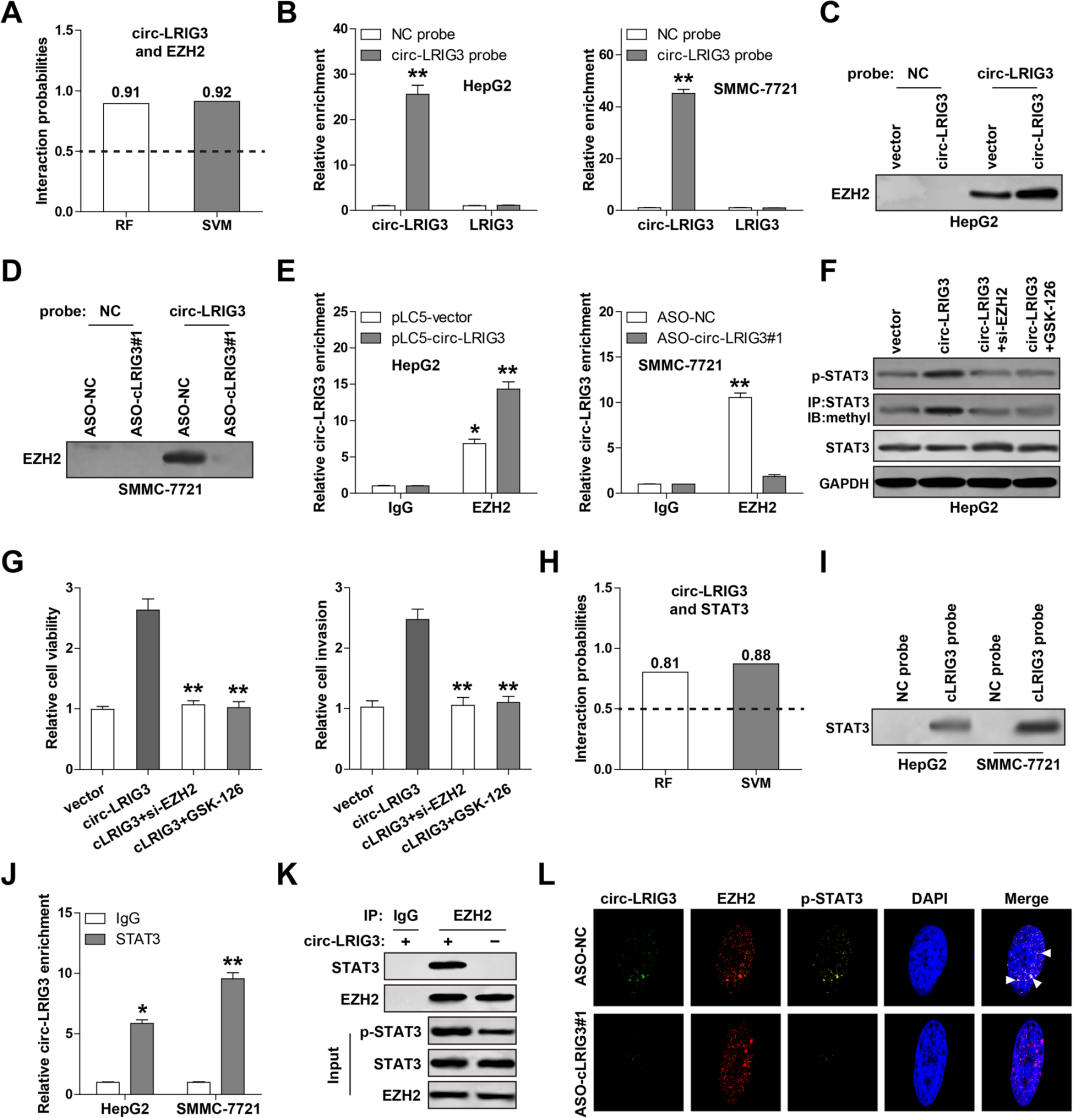
Circ-LRIG3 forms a ternary with EZH2 and STAT3. **a** The interaction probability between circ-LRIG3 and EZH2 predicted by RPISeq online software based on RF and SVM classifiers (Interaction probabilities generated by RPISeq range from 0 to 1. In performance evaluation experiments, predictions with probabilities > 0.5 were considered “positive.”). **b** qRT-PCR analysis verifying the efficiency of circ-LRIG3 probe. **c**, **d** RNA pull-down assay using circ-LRIG3 probe coupled Western blot analysis of EZH2 protein expression. **e** RIP assay using anti-EZH2 antibody coupled qRT-PCR analysis of circ-LRIG3 enrichment. **f** Western blot analysis of the indicated protein levels in circ-LRIG3-overexpressing HepG2 cells treated with EZH2 siRNA or GSK-126. **g** CCK-8 and Transwell assays detecting the cell viability and invasion in circ-LRIG3-overexpressing HepG2 cells treated with EZH2 siRNA or GSK-126. **h** RPISeq online software predicting the interaction probability between circ-LRIG3 and STAT3. **i**, **j** RNA pull-down assay using circ-LRIG3 probe and RIP assay using anti-STAT3 antibody in HepG2 and SMMC-7721 cells. **k** Co-IP assay in circ-LRIG3-silenced SMMC-7721 cells using anti-EZH2 antibody, followed by Western blot analysis of EZH2 and STAT3 protein levels. **l** The co-location between circ-LRIG3, EZH2 and p-STAT3. *P < 0.05, **P < 0.01

Given that EZH2 can directly bind STAT3 to increase STAT3 methylation and subsequent phosphorylation [18], we wondered whether circ-LRIG3 activates STAT3 through EZH2. The RPISeq online tool result showed that circ-LRIG3 and EZH2 are highly likely to interact (RF classifier = 0.91, SVM classifier = 0.92) (http://pridb.gdcb.iastate.edu/) (Fig. 4a). We then designed biotinylated circ-LRIG3 probe to perform RNA pull-down assay, the circ-LRIG3 probe was verified to effectively enrich circ-LRIG3, but not linear LRIG3 (Fig. 4b). As shown in Fig. 4c, EZH2 was abundantly enriched by circ-LRIG3 probe in HepG2 cells, and this enrichment was significantly increased by circ-LRIG3 overexpression. Likewise, the interaction between circ-LRIG3 and EZH2 was also observed in SMMC-7721 cells, and circ-LRIG3 depletion evidently decreased this phenomenon (Fig. 4d). To confirm above results, we performed RNA immunoprecipitation (RIP) assay, and found that circ-LRIG3 was significantly immunoprecipitated by anti-EZH2 antibody as compared with negative anti-IgG antibody (Fig. 4e). Importantly, circ-LRIG3 overexpression increased STAT3 methylation and phosphorylation, whereas these effects were completely blocked after treatment with EZH2 siRNA or GSK-126, a highly selective EZH2 inhibitor (Fig. 4f). Similarly, EZH2 siRNA or GSK-126 effectively abrogated the enhanced malignant phenotype induced by circ-LRIG3 overexpression in HepG2 cells (Fig. 4g). These indicate that EZH2 is required for circ-LRIG3-induced STAT3 activation. In additions, the RPISeq online tool also showed that circ-LRIG3 and STAT3 are highly likely to interact (RF classifier = 0.81, SVM classifier = 0.88) (Fig. 4h), and this prediction was confirmed by RNA pull-down assay using biotin-labeled circ-RIG3 probe (Fig. 4i) and RIP assay using anti-STAT3 antibody (Fig. 4j) in both HepG2 and SMMC-7721 cells. More importantly, the Co-IP results showed that endogenous EZH2 and STAT3 were directly bound, whereas this interaction was almost disappeared after circ-LRIG3 knockdown (Fig. 4k), suggesting that circ-LRIG3 is critical for the binding between EZH2 and STAT3. And the immunofluorescence (IF) results showed that circ-LRIG3 was co-located with EZH2 and p-STAT3 in the nucleus (Fig. 4l), and this phenomenon was decreased by circ-LRIG3 depletion (Fig. 4l).

### Circ-LRIG3 was transcriptionally regulated by activated STAT3

**Fig. 5:**
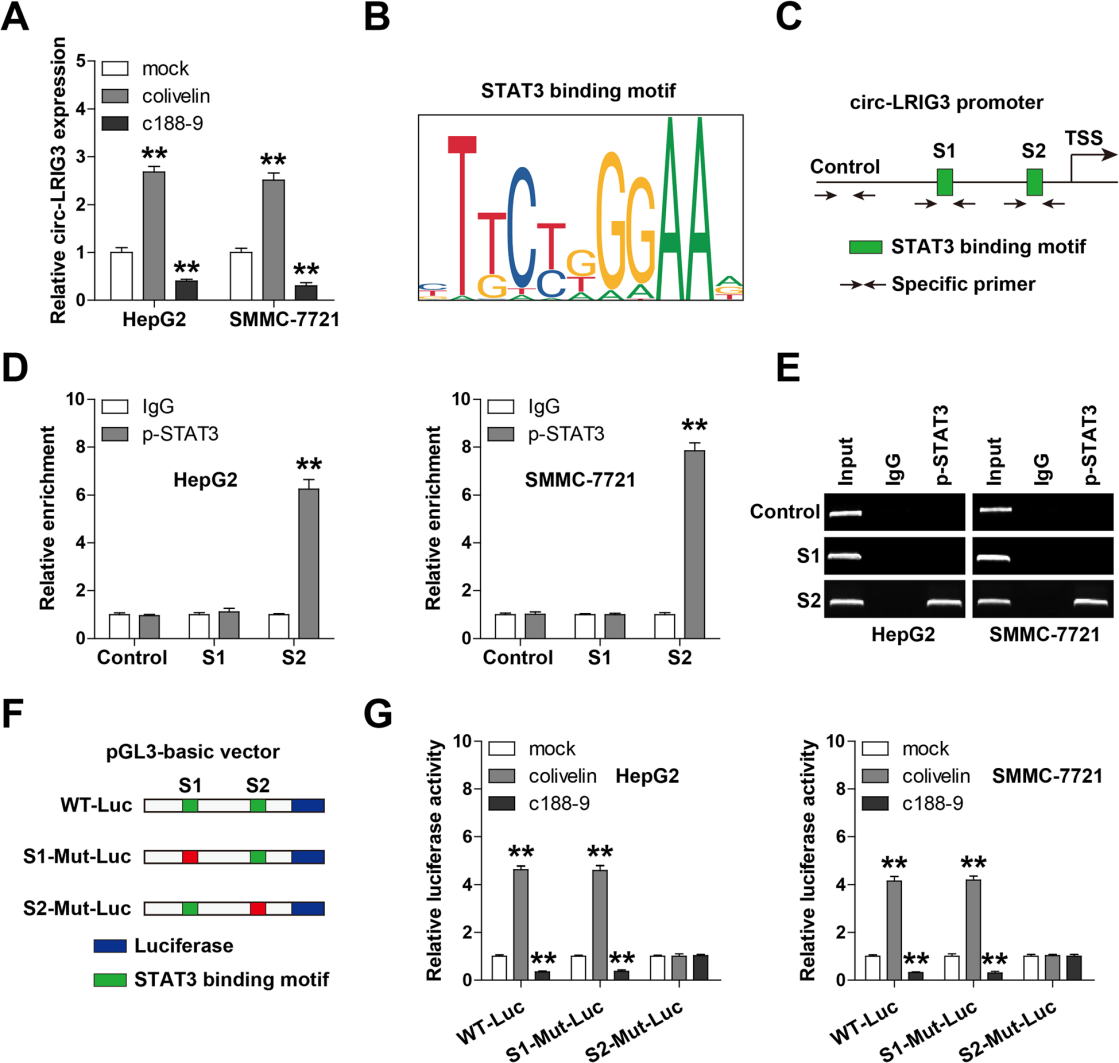
Circ-LRIG3 is regulated by p-STAT3. **a** qRT-PCR analysis of circ-LRIG3 in HepG2 and SMMC-7721 cells treated with colivelin and C188–9. **b** The STAT3 binding motif on circ-LRIG3 promoter predicted by JASPAR online tool. **c** The designed primers targeting the STAT3 binding sites on circ-LRIG3 promoter used for ChIP assay. **d**, **e** ChIP assay using anti-p-STAT3 antibody, followed by PCR analysis of enrichment. Anti-IgG antibody was used as a negative control. **f** The sketch showing the pGL3-basic reporter containing full-length circ-LRIG3 promoter sequence with wild-type or mutated STAT3 binding motif. **g** Luciferase reporter assay in HepG2 and SMMC-7721 cells transfected with above wild-type or mutated pGL3-basic reporter. **P < 0.01

Given that STAT3 is a critical transcription factor, we then test whether circ-LRIG3 is also regulated by STAT3. After treatment of HepG2 and SMMC-7721 cells with colivelin, a specific STAT3 activator, the expression levels of circ-LRIG3 were remarkably increased, while treatment with C188–9 resulted in an opposite effect (Fig. 5a). Through analyzing the JASPAR database, two STAT3 binding motifs were found on circ-LRIG3 promoter (Fig. 5b, c). We then performed chromatin immunoprecipitation (ChIP) coupled PCR assay, the results showed that p-STAT3 was abundantly enriched on site 2 adjacent to the transcription initiation site (TSS), but not on site 1 and negative control region (Fig. 5d, e). Further, we performed luciferase reporter assay using pGL3-basic vector with wild-type or mutant STAT3 binding site (Fig. 5f), the results showed that colivelin and C188–9 significantly increased and decreased wild-type circ-LRIG3 transcriptional activity, respectively, whereas mutant of site 2, not site 1, completely blocked above effects (Fig. 5g). These data reveal that p-STAT3 directly binds to circ-LRIG3 promoter and activates its transcription.

### Exogenous expression of circ-LRIG3 enhances tumorigenicity and metastasis in vivo, which is effectively counteracted by C188–9

**Fig. 6:**
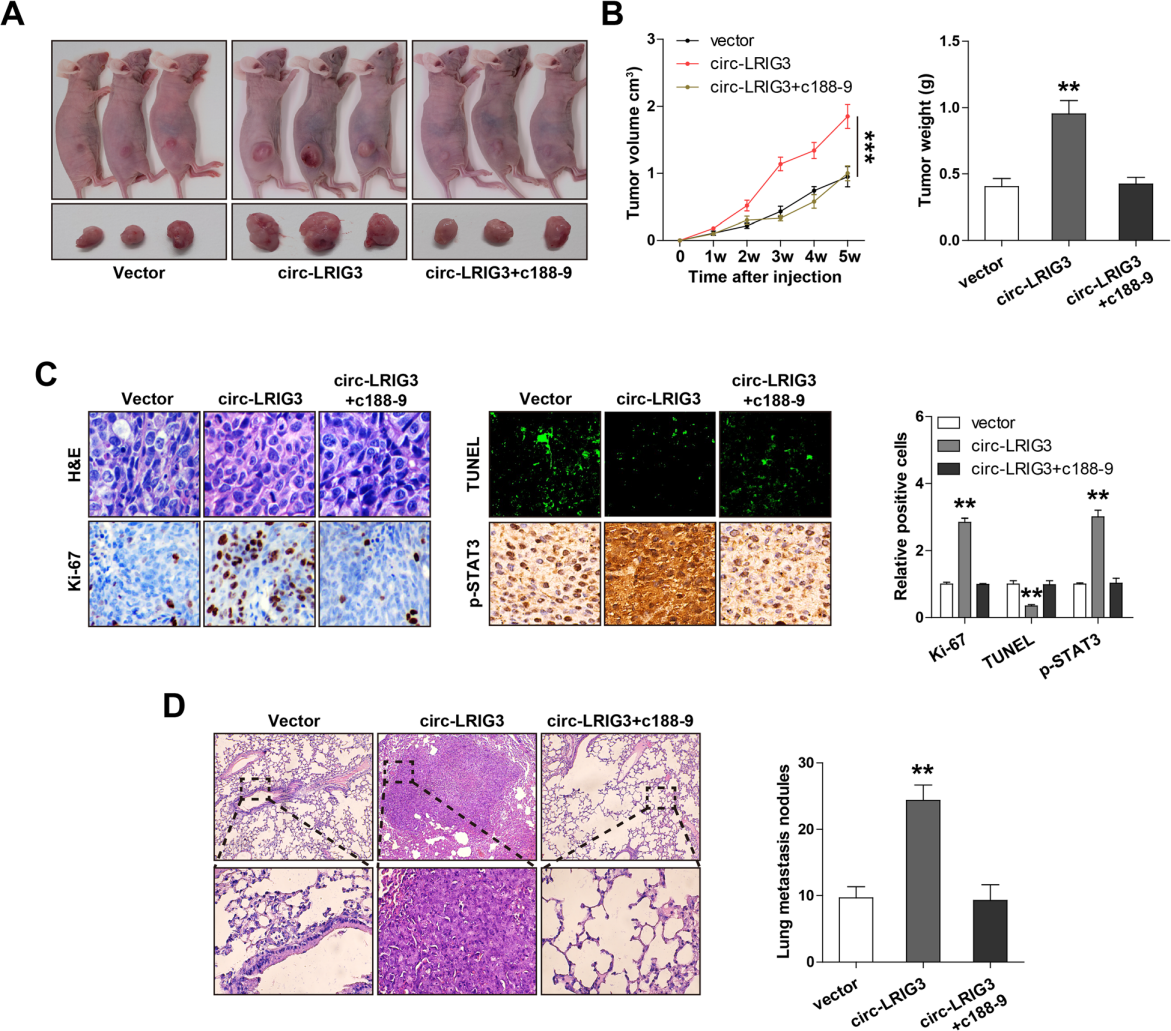
Circ-LRIG3 is a oncogenic circRNA in vivo. **a**, **b** The image, volume weight of mice bearing tumors of circ-LRIG3-overexpressing cells treated with C188–9 (50 mg/kg) (*n* = 3 in per group). **c** H&E, IHC and TUNEL staining in tumor tissues of nude mice from the indicated three groups. **d** H&E staining of lung tissues in the indicated three groups (*n* = 3 in per group). **P < 0.01,***P < 0.001

Lastly, we tested whether circ-LRIG3 was functional in vivo via subcutaneous injection of HepG2 cells into nude mice. As shown in Fig. 6a, b, mice bearing tumors of circ-LRIG3-overexpressing cells had larger tumor volume and weight than mice bearing tumors of control cells. Moreover, more Ki-67 and p-STAT3 positive cells, while less apoptotic cells were observed in circ-LRIG3-overexpressing group as compared with control group (Fig. 6c). Further, we established lung metastasis model by caudal vein injection of circ-LRIG3-overexpressing HepG2 cells into nude mice, the results showed that circ-LRIG3 overexpression notably increased the number of lung metastasis nodules (Fig. 6d). Importantly, when nude mice were treated with C188–9, the increased tumor size and lung metastasis nodules caused by circ-LRIG3 overexpression were restored to control levels (Fig. 6a-d).

## Discussion

**Fig. 7:**
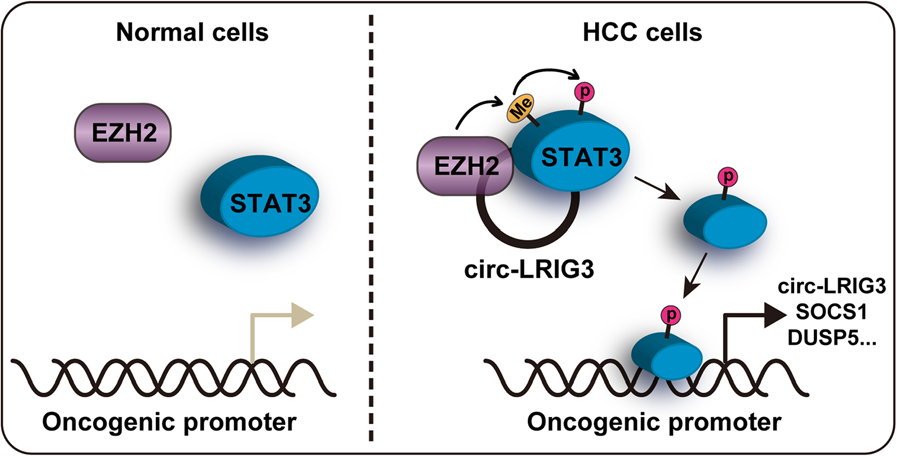
The proposed model showing that EZH2 binds to, methylates and phosphorylates STAT3 in the presence of circ-LRIG3, resulting in the activation of STAT3 signaling, promoting HCC tumorigenesis and progression

Emerging evidence suggest that circRNA is frequently dysregulated in human cancer, and affects tumorigenesis, relapse and dissemination through regulating different signaling pathways [19, 20]. In our previous study, we identified a large number of dysregulated circRNAs in HCC via performing microarray expression profile [17], and found that circ-ADD3 served as a metastasis-inhibiting circRNA through degradation of EZH2 [17]. In the present study, we described the role of circ-LRIG3 in HCC. Circ-LRIG3 was a nuclear circRNA that was notably increased in HCC tissues, cells and plasma. It physically interacted with EZH2 and STAT3 and acted as a scaffold to increase STAT3 methylation and subsequent phosphorylation induced by EZH2; in turn, activated STAT3 directly bound onto circ-LRIG3 promoter region to enhance circ-LRIG3 transcriptional activity, thus forming a positive regulatory circuit to promote the progression of HCC (Fig. 7). Therefore, our data uncover the oncogenic effect of circ-LRIG3 in HCC, meanwhile provide new evidence for the important role of circRNA in cancer cell biology.

Because of its special covalent ring structure resisting to exonuclease, circRNA is extraordinary stable, enabling it to be used as an excellent disease marker [21–23]. For instance, a recent study performed the RNA-seq analysis of 20 paired frozen tissues collected postoperation, and found that four-circRNAs (hsa_circ_0122319, hsa_circ_0087391, hsa_circ_0079480 and hsa_circ_0008039)-based cirScore could reliably predict postoperative recurrence in stage II/III colon cancer [24]. Besides, circ-ANKS1B was reported to be significantly upregulated in triple-negative breast cancer, and it was a high risk predictor of breast cancer metastasis [25]. Herein, we found that circ-LRIG3 was notably increased in HCC and patients with high circ-LRIG3 had shorter overall and disease-free survival time than those with low circ-LRIG3, implying that circ-LRIG3 is a prognostic biomarker of HCC. Further, we found that circ-LRIG3 was also upregulated in HCC plasma, the AUC value was 0.8681 (95%CI: 0.7843 ~ 0.9519), suggesting its great potential as a non-invasive diagnostic marker of HCC. Of note, recent studies show that some circRNAs are enriched and stable in exosomes [26, 27], such as hsa_circ_0051443 [28], hsa_circ_100,338 [29] and hsa_circ_0006156 [30]. Whether circ-LRIG3 also exists in exosomes is worth further study, in addition, the multi-center large-scale sample investigation is necessary to confirm the diagnostic and prognostic value of circ-LRIG3 in HCC.

Numerous studies suggest that ncRNA can directly interact with functional proteins to shape cellular activity [31]. The dysregulation of ncRNA (especially long non-coding RNA, lncRNA)-protein interaction network has recently been proved to be critical for cancer development and progression [32]. However, there is little evidence showing that circRNA functions by interacting with proteins. In the present study, through performing RNA pull-down and RIP assays, we found that circ-LRIG3 was able to bind EZH2 in the nucleus. EZH2 is a well-known oncogene that can interact with different ncRNAs, such as HERES [33], LINC00673 [34] and SPRY4-IT1 [35]. Similarly, our research team previously found that EZH2 could directly bind circ-ADD3 and its protein expression was reduced by circ-ADD3 [17]. However, here, we found that the binding between circ-LRIG3 and EZH2 did not affect EZH2 expression, but promoted the methylation and phosphorylation of STAT3. Of note, EZH2-induced STAT3 activation was disappeared in the absence of circ-LRIG3, indicating that circ-LRIG3 serves as a scaffold for the interaction between EZH2 and STAT3. Further research is needed to determine which regions are necessary for the formation of this circ-LRIG3/EZH2/STAT3 ternary complex, so as to design small interfering peptides to block their binding, which may be a promising new approach for the treatment of HCC.

The STAT3 signaling pathway is oncogenic and frequently over-activated in various human cancer types, including HCC [36]. Phosphorylated STAT3 can form a transcriptionally active STAT3-STAT3 dimer, followed by binding to specific motifs on gene promoters in the nucleus to regulate gene expression, such as SOCS1 and DUSP5 [37]. In this study, we also found two STAT3 binding motifs on circ-LRIG3 promoter. Through performing ChIP-PCR and luciferase reporter assays, we found that activated STAT3 could bind to the motifs adjacent to the TSS, resulting in the activation of circ-LRIG3 transcription, thus they form a positive feedback loop, amplifying the carcinogenic effect of circ-LRIG3 in HCC.

## Conclusions

In sum, our findings for the first time demonstrate that circ-LRIG3 is a tumor-promoting circRNA in HCC, it promotes hepatocarcinogenesis and metastasis through acting as a protein binding partner. Therefore, we provide a potential diagnostic, prognostic and therapeutic target for patients with HCC.

## Supplementary Information

**Additional file 1.**

**Additional file 2.**

## Data Availability

The dataset used and/or analyzed during the current study are available from the corresponding author on reasonable request.

## Abbreviations

circRNA: Circular RNA
HCC: Hepatocellular carcinoma
ncRNA: Non coding RNA
ASO: Antisense oligonucleotide
IHC: Immunohistochemistry
GSEA: Gene Set Enrichment Analysis
ChIP: Chromatin immunoprecipitation
H&E: Hematoxylin&eosin

## References

[1] McGlynn KA, Petrick JL, El-Serag HB. Epidemiology of Hepatocellular Carcinoma. Hepatology. 2020; 10.1002/hep.31288. 10.1002/hep.31288 PMC7577946 32319693

[2] Mullath A, Krishna M (2019). Hepatocellular carcinoma - time to take the ticket. World J Gastrointest Surg.

[3] Lu LC, Cheng AL, Poon RT (2014). Recent advances in the prevention of hepatocellular carcinoma recurrence. Semin Liver Dis.

[4] Bruix J, Gores GJ, Mazzaferro V (2014). Hepatocellular carcinoma: clinical frontiers and perspectives. Gut..

[5] Wilusz JE (2018). A 360 degrees view of circular RNAs: from biogenesis to functions. Wiley Interdiscip Rev RNA.

[6] Salzman J, Gawad C, Wang PL, Lacayo N, Brown PO (2012). Circular RNAs are the predominant transcript isoform from hundreds of human genes in diverse cell types. PLoS One.

[7] Wang PL, Bao Y, Yee MC, Barrett SP, Hogan GJ, Olsen MN, Dinneny JR, Brown PO, Salzman J (2014). Circular RNA is expressed across the eukaryotic tree of life. PLoS One.

[8] Kristensen LS, Andersen MS, Stagsted L, Ebbesen KK, Hansen TB, Kjems J (2019). Nat Rev Genet.

[9] Jeck WR, Sorrentino JA, Wang K, Slevin MK, Burd CE, Liu J, Marzluff WF, Sharpless NE (2013). Circular RNAs are abundant, conserved, and associated with ALU repeats. Rna..

[10] Tay Y, Rinn J, Pandolfi PP (2014). The multilayered complexity of ceRNA crosstalk and competition. Nature..

[11] Thomson DW, Dinger ME (2016). Endogenous microRNA sponges: evidence and controversy. Nat Rev Genet.

[12] Memczak S, Jens M, Elefsinioti A, Torti F, Krueger J, Rybak A, Maier L, Mackowiak SD, Gregersen LH, Munschauer M (2013). Circular RNAs are a large class of animal RNAs with regulatory potency. Nature..

[13] Hansen TB, Jensen TI, Clausen BH, Bramsen JB, Finsen B, Damgaard CK, Kjems J (2013). Natural RNA circles function as efficient microRNA sponges. Nature..

[14] Du WW, Zhang C, Yang W, Yong T, Awan FM, Yang BB (2017). Identifying and characterizing circRNA-protein interaction. Theranostics..

[15] Yang Q, Du WW, Wu N, Yang W, Awan FM, Fang L, Ma J, Li X, Zeng Y, Yang Z (2017). A circular RNA promotes tumorigenesis by inducing c-myc nuclear translocation. Cell Death Differ.

[16] Li H, Yang F, Hu A, Wang X, Fang E, Chen Y, Li D, Song H, Wang J, Guo Y (2019). Therapeutic targeting of circ-CUX1/EWSR1/MAZ axis inhibits glycolysis and neuroblastoma progression. Embo Mol Med.

[17] Sun S, Wang W, Luo X, Li Y, Liu B, Li X, Zhang B, Han S, Li X (2019). Circular RNA circ-ADD3 inhibits hepatocellular carcinoma metastasis through facilitating EZH2 degradation via CDK1-mediated ubiquitination. Am J Cancer Res.

[18] Kim E, Kim M, Woo DH, Shin Y, Shin J, Chang N, Oh YT, Kim H, Rheey J, Nakano I (2013). Phosphorylation of EZH2 activates STAT3 signaling via STAT3 methylation and promotes tumorigenicity of glioblastoma stem-like cells. Cancer Cell.

[19] Bach DH, Lee SK, Sood AK (2019). Circular RNAs in Cancer. Mol Ther Nucleic Acids.

[20] Wu J, Qi X, Liu L, Hu X, Liu J, Yang J, Yang J, Lu L, Zhang Z, Ma S (2019). Emerging epigenetic regulation of circular RNAs in human Cancer. Mol Ther Nucleic Acids.

[21] Vo JN, Cieslik M, Zhang Y, Shukla S, Xiao L, Zhang Y, Wu YM, Dhanasekaran SM, Engelke CG, Cao X (2019). The landscape of circular RNA in Cancer. Cell..

[22] Zhang Z, Yang T, Xiao J (2018). Circular RNAs: promising biomarkers for human diseases. Ebiomedicine..

[23] Zhang Y, Liang W, Zhang P, Chen J, Qian H, Zhang X, Xu W (2017). Circular RNAs: emerging cancer biomarkers and targets. J Exp Clin Cancer Res.

[24] Ju HQ, Zhao Q, Wang F, Lan P, Wang Z, Zuo ZX, Wu QN, Fan XJ, Mo HY, Chen L (2019). A circRNA signature predicts postoperative recurrence in stage II/III colon cancer. Embo Mol Med.

[25] Zeng K, He B, Yang BB, Xu T, Chen X, Xu M, Liu X, Sun H, Pan Y, Wang S (2018). The pro-metastasis effect of circANKS1B in breast cancer. Mol Cancer.

[26] Li Y, Zheng Q, Bao C, Li S, Guo W, Zhao J, Chen D, Gu J, He X, Huang S (2015). Circular RNA is enriched and stable in exosomes: a promising biomarker for cancer diagnosis. Cell Res.

[27] Wang Y, Liu J, Ma J, Sun T, Zhou Q, Wang W, Wang G, Wu P, Wang H, Jiang L (2019). Exosomal circRNAs: biogenesis, effect and application in human diseases. Mol Cancer.

[28] Chen W, Quan Y, Fan S, Wang H, Liang J, Huang L, Chen L, Liu Q, He P, Ye Y (2020). Exosome-transmitted circular RNA hsa_circ_0051443 suppresses hepatocellular carcinoma progression. Cancer Lett.

[29] Huang XY, Huang ZL, Huang J, Xu B, Huang XY, Xu YH, Zhou J, Tang ZY (2020). Exosomal circRNA-100338 promotes hepatocellular carcinoma metastasis via enhancing invasiveness and angiogenesis. J Exp Clin Cancer Res.

[30] Wu G, Zhou W, Pan X, Sun Z, Sun Y, Xu H, Shi P, Li J, Gao L, Tian X (2020). Circular RNA profiling reveals Exosomal circ_0006156 as a novel biomarker in papillary thyroid Cancer. Mol Ther Nucleic Acids.

[31] Slack FJ, Chinnaiyan AM (2019). The role of non-coding RNAs in oncology. Cell..

[32] Sauvageau M (2019). Diverging RNPs: toward understanding lncRNA-protein interactions and functions. Adv Exp Med Biol.

[33] You BH, Yoon JH, Kang H, Lee EK, Lee SK, Nam JW (2019). HERES, a lncRNA that regulates canonical and noncanonical Wnt signaling pathways via interaction with EZH2. Proc Natl Acad Sci U S A.

[34] Huang M, Hou J, Wang Y, Xie M, Wei C, Nie F, Wang Z, Sun M (2017). Long noncoding RNA LINC00673 is activated by SP1 and exerts oncogenic properties by interacting with LSD1 and EZH2 in gastric Cancer. Mol Ther.

[35] Xu Y, Yao Y, Jiang X, Zhong X, Wang Z, Li C, Kang P, Leng K, Ji D, Li Z (2018). SP1-induced upregulation of lncRNA SPRY4-IT1 exerts oncogenic properties by scaffolding EZH2/LSD1/DNMT1 and sponging miR-101-3p in cholangiocarcinoma. J Exp Clin Cancer Res.

[36] Subramaniam A, Shanmugam MK, Perumal E, Li F, Nachiyappan A, Dai X, Swamy SN, Ahn KS, Kumar AP, Tan BK (1835). Potential role of signal transducer and activator of transcription (STAT)3 signaling pathway in inflammation, survival, proliferation and invasion of hepatocellular carcinoma. Biochim Biophys Acta.

[37] Yu H, Pardoll D, Jove R (2009). STATs in cancer inflammation and immunity: a leading role for STAT3. Nat Rev Cancer.

