# Supplementary material for: A novel circular RNA circ-LRIG3 facilitates the malignant progression of hepatocellular carcinoma by modulating the EZH2/STAT3 signaling
Source: J Exp Clin Cancer Res. 2020 Nov 23;39:252. doi: 10.1186/s13046-020-01779-5 (PMC7682056; doi:10.1186/s13046-020-01779-5)
Supplement: Supplementary file 1 — Additional file 1. [file 13046_2020_1779_MOESM1_ESM.docx]

**Table S1.** Correlation between circ-LRIG3 expression and clinicopathologic features of HCC patients (n=130)

| Features | circ-LRIG3 expression | | *P* value |
| --- | --- | --- | --- |
|  | Low (n=65) | High (n=65) |  |
| Age |  |  | 0.725 |
| ≤ 50 | 31 | 29 |  |
| > 50 | 34 | 36 |  |
| Gender |  |  | 0.804 |
| Male | 56 | 55 |  |
| Female | 9 | 10 |  |
| AFP (ng/ml) |  |  | 0.175 |
| ≤ 400 | 15 | 9 |  |
| > 400 | 50 | 56 |  |
| Tumor diameter (cm) |  |  | 0.001 |
| ≤ 5 | 38 | 19 |  |
| > 5 | 27 | 46 |  |
| Vascular invasion |  |  | 0.000 |
| No | 24 | 5 |  |
| Yes | 41 | 60 |  |
| Edmondson’s grade |  |  | 0.008 |
| I-II | 27 | 13 |  |
| III-IV | 38 | 52 |  |
| TNM stage |  |  | 0.002 |
| I-II | 32 | 15 |  |
| III-IV | 33 | 50 |  |

**Table S2**. Uni- and multivariate analysis of prognostic predictors for overall survival in HCC patients (n=130)

| Variable | Univariate analysis |  | Multivariate  analysis |  |
| --- | --- | --- | --- | --- |
|  | HR (95% CI) | *P* value | HR (95% CI) | *P* value |
| Age | - | 0.785 | - | - |
| Gender | - | 0.563 | - | - |
| AFP | 3.423 (1.521-3.785) | 0.003 | - | 0.365 |
| Tumor diameter | 2.117 (1.285-3.681) | 0.012 | - | 0.174 |
| Vascular invasion | 3.562 (2.085-5.741) | 0.005 | 2.347 (1.856-4.015) | 0.018 |
| Edmondson’s grade | 2.314 (2.011-6.358) | 0.023 | - | 0.415 |
| TNM stage | 6.534 (2.885-9.724) | 0.000 | 3.245 (2.702-5.261) | 0.031 |
| circ-LRIG3 | 5.116 (1.892-7.452) | 0.000 | 4.774 (2.351-8.068) | 0.009 |

**Table S3**. The primer sequences used in this study

| **Gene** | **Direction** | **Sequence** |
| --- | --- | --- |
| circ-LRIG3 (divergent) | Forward | TCACTGGTTTGGATGCATTG |
|  | Reverse | AAGGTGGCTCATGGAACTTG |
| circ-LRIG3 (convergent) | Forward | GGACCTAACTTTCAATCACTTATCAA |
|  | Reverse | CGGAAGGCACAATCAGCA |
| LRIG3 | Forward | GCGAACGGAGCTTCAGTAAT |
|  | Reverse | TGGCTCTGGATTTGCACTAA |
| FAS | Forward | AATGCCCAAGTGACTGACATC |
|  | Reverse | GACAGGGCTTATGGCAGAAT |
| SOCS1 | Forward | GAGCTGCTGGAGCACTACG |
|  | Reverse | AGGGGAAGGAGCTCAGGTAG |
| DUSP5 | Forward | CTTGGAAGTGCCTACCATGC |
|  | Reverse | GGAGCTAATGTCAGCCGTGT |
| CXCL1 | Forward | AGGGAATTCACCCCAAGAAC |
|  | Reverse | TAACTATGGGGGATGCAGGA |
| STAM2 | Forward | CGCTGCAAGAACAGAAACAG |
|  | Reverse | TCATTGTCCTCAACAGCTTCA |
| GAPDH | Forward | ACCCAGAAGACTGTGGATGG |
|  | Reverse | TTCAGCTCAGGGATGACCTT |
