# Supplementary material for: A novel circular RNA circ-LRIG3 facilitates the malignant progression of hepatocellular carcinoma by modulating the EZH2/STAT3 signaling
Source: J Exp Clin Cancer Res. 2020 Nov 23;39:252. doi: 10.1186/s13046-020-01779-5 (PMC7682056; doi:10.1186/s13046-020-01779-5)
Supplement: Supplementary file 2 — Additional file 2. [file 13046_2020_1779_MOESM2_ESM.doc]

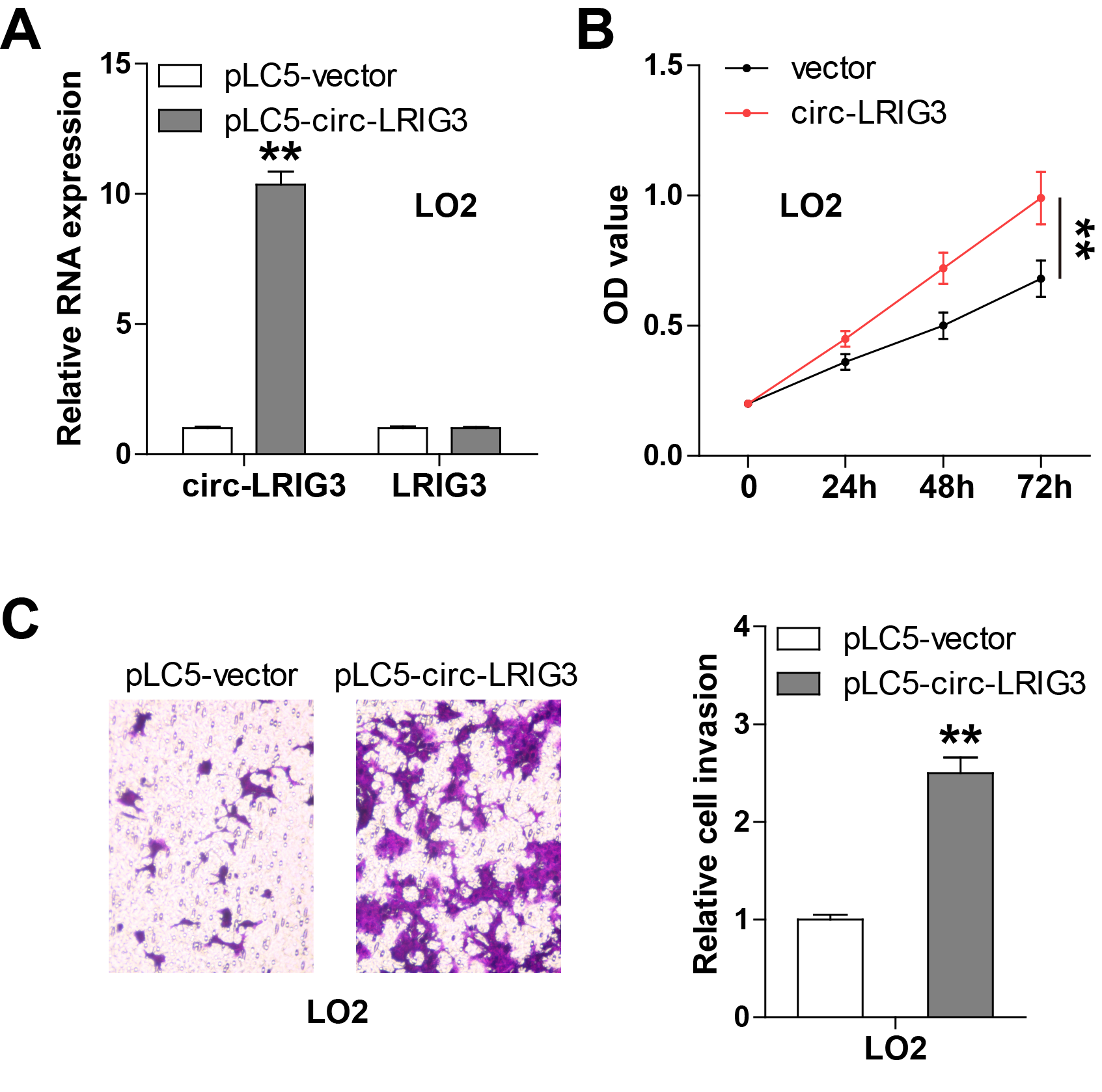


**Figure S1: Circ-LRIG3 promotes LO2 cell viability and invasion.** A. qRT-PCR analysis of circ-LRIG3 and LRIG3 mRNA levels in LO2 cells tansfected with the indicated vectors. B. CCK-8 assay detecting cell viability in LO2 cells after circ-LRIG3 overexpression. C. Transwell assay detecting cell invasion in LO2 cells after circ-LRIG3 overexpression. ^**^ *P*<0.01.
